# Discontinuous peripheral enhancement of focal liver lesions on CT and MRI: outside the box of typical cavernous hemangioma

**DOI:** 10.1007/s00261-024-04522-2

**Published:** 2024-08-27

**Authors:** Francesco Matteini, Roberto Cannella, Marco Dioguardi Burgio, Chiara Torrisi, Riccardo Sartoris, Giuseppe Brancatelli, Valérie Vilgrain, Maxime Ronot, Federica Vernuccio

**Affiliations:** 1 Department of Biomedicine, Neuroscience and Advanced Diagnostics (Bi.N.D.), University Hospital of Palermo, Via del Vespro 129, 90127 Palermo, Italy; 2 Department of Health Promotion, Mother and Child Care, Internal Medicine and Medical Specialties (PROMISE), University of Palermo, Via del Vespro, 129, 90127 Palermo, Italy; 3 Department of Radiology, Hôpital Beaujon, AP-HP.Nord, Paris, France; 4 INSERM U1149 Centre de Recherche sur l’Inflammation (CRI), Université Paris Cité, Paris, France; 5 IRCCS Humanitas Research Hospital, Via Manzoni 56, Rozzano, 20089 Milan, Italy

**Keywords:** Liver imaging, CT, Magnetic resonance imaging, Contrast agent intravenous, Haemangioma, Hepatic infection, Liver neoplasm, Hepatocellular carcinoma, Liver metastases

## Abstract

The discontinuous peripheral enhancement is a pattern of enhancement usually attributed to typical cavernous hemangioma, that is the most common benign solid lesion of the liver. The discontinuous peripheral enhancement, however, may be encountered in many other benign and malignant focal liver lesions as an atypical presentation or evolution, and hemangiomas with discontinuous peripheral hyperenhancement on hepatic arterial phase may not always have the typical post-contrast pattern on portal venous and delayed phases. Therefore, abdominal radiologists may be challenged in their practice by lesions with discontinuous peripheral enhancement. This pictorial essay aims to review the spectrum of benign and malignant focal liver lesions that may show discontinuous peripheral enhancement. A particular point of interest is the diagnostic tree pathway that may guide the radiologists in the differential diagnosis.

**Graphical abstract:**

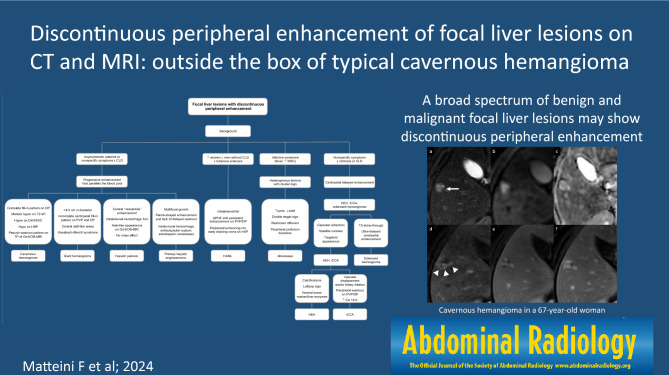

## Introduction

Focal liver lesions with peripheral enhancement on contrast-enhanced computed tomography (CT) and magnetic resonance imaging (MRI), include a broad range of entities that may demonstrate discontinuous peripheral enhancement, rim enhancement or corona enhancement on late hepatic arterial phase [1]. Among these, the discontinuous peripheral enhancement, also known as peripheral nodular enhancement, is a pattern of enhancement usually attributed to typical cavernous hemangiomas [2]. Cavernous hemangioma is the most common benign solid lesion of the liver and typically demonstrates peripheral discontinuous nodular enhancement on hepatic arterial phase (HAP), followed by continued centripetal “filling-in” and persistent enhancement that parallels blood pool in the portal venous phase (PVP) and delayed phase (DP) [3, 4]. The identification of all these enhancement characteristics allows a confident diagnosis of cavernous hemangioma, thus indicating the lack of need for any follow-up or treatment [5]. However, some atypical hemangiomas may show only peripheral discontinuous nodular enhancement on arterial phase and lack the remaining characteristics; in addition, other benign and malignant liver lesions may show a discontinuous peripheral enhancement that may mimic a cavernous hemangioma.

The multifaceted presentation of focal liver lesions with discontinuous peripheral enhancement on contrast-enhanced CT and MRI may challenge the radiologists in the interpretation of exams. The evaluation of the clinical setting (e.g. fever), history of the patient (e.g. presence or not of chronic hepatopathy) and laboratory findings (e.g. increased C-reactive protein) represents the first step in the assessment of focal liver lesions on CT/MRI images to make the correct diagnosis. Cross-sectional imaging plays a pivotal role in the differential diagnosis, particularly in identifying lesions which need no treatment, medical therapy, or invasive approaches. This pictorial essay is aimed at providing a critical review of the essential, and optional imaging reporting elements used to differentiate focal liver lesions with discontinuous peripheral enhancement. A particular point of interest is the diagnostic tree pathway provided as a guide for the differential diagnosis.

## Benign lesions

### Vascular

#### Hemangioma

**Fig. 1:**
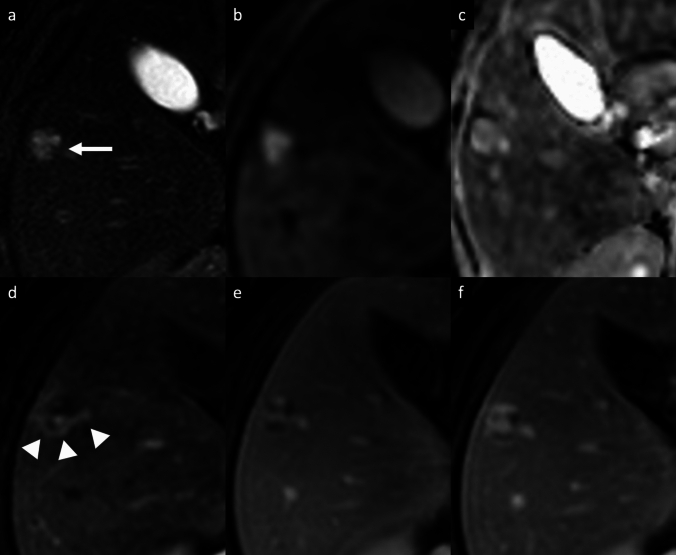
Cavernous hemangioma in a 67-year-old woman with hepatic steatosis. Magnetic resonance images show a lobulated focal liver lesion in the right hepatic lobe with high signal intensity on fat-suppressed T2-weighted image (arrow, **a**), high signal intensity on diffusion-weighted images (**b**) and high signal intensity on ADC map (**c**). On extracellular contrast agent-enhanced MRI, the lesion demonstrates peripheral discontinuous nodular enhancement on arterial phase (arrowheads, **d**), followed by progressive centripetal incomplete enhancement on portal venous (**e**) and delayed phases (**f**)

**Fig. 2:**
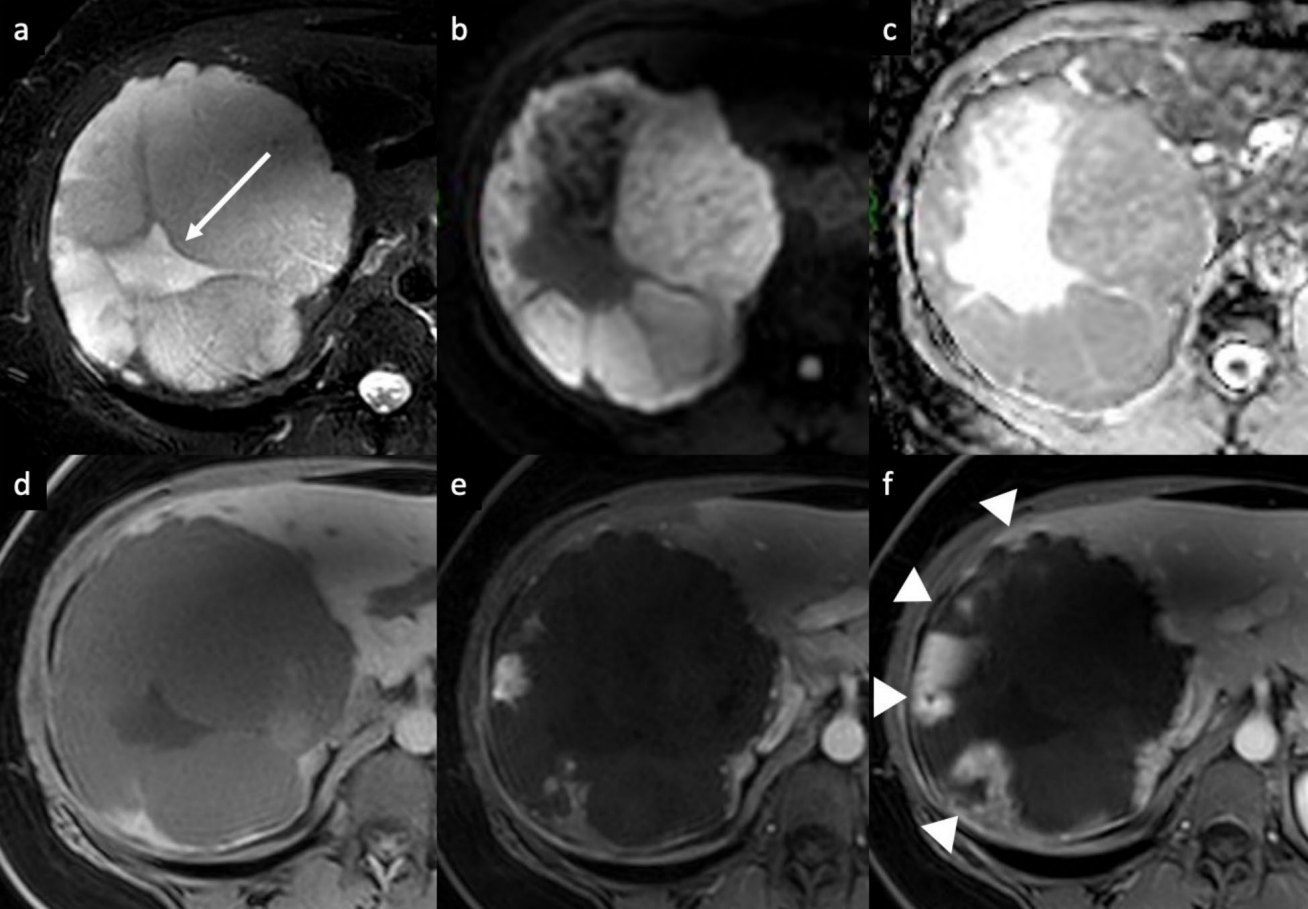
Giant hemangioma in a 40-year-old man who presented with abdominal pain. **a** Axial fat-suppressed T2-weighted MRI shows a large, lobulated, markedly hyperintense liver lesion located in the right hepatic lobe, with high signal intensity cleftlike area (arrow) and some hypointense internal septa. The lesion shows high signal intensity on diffusion-weighted images (DWI) (**b**) and high signal intensity on apparent diffusion coefficient (ADC) map (**c**); the cleftlike area and the internal septa appear hypointense on DWI, with marked high signal intensity on ADC map. The lesion displays a low signal intensity on unenhanced T1-weighted image (**d**), with peripheral discontinuous nodular enhancement on arterial phase (**e**), and progressive but incomplete peripheral filling on delayed phase (arrowheads, **f**)

**Fig. 3:**
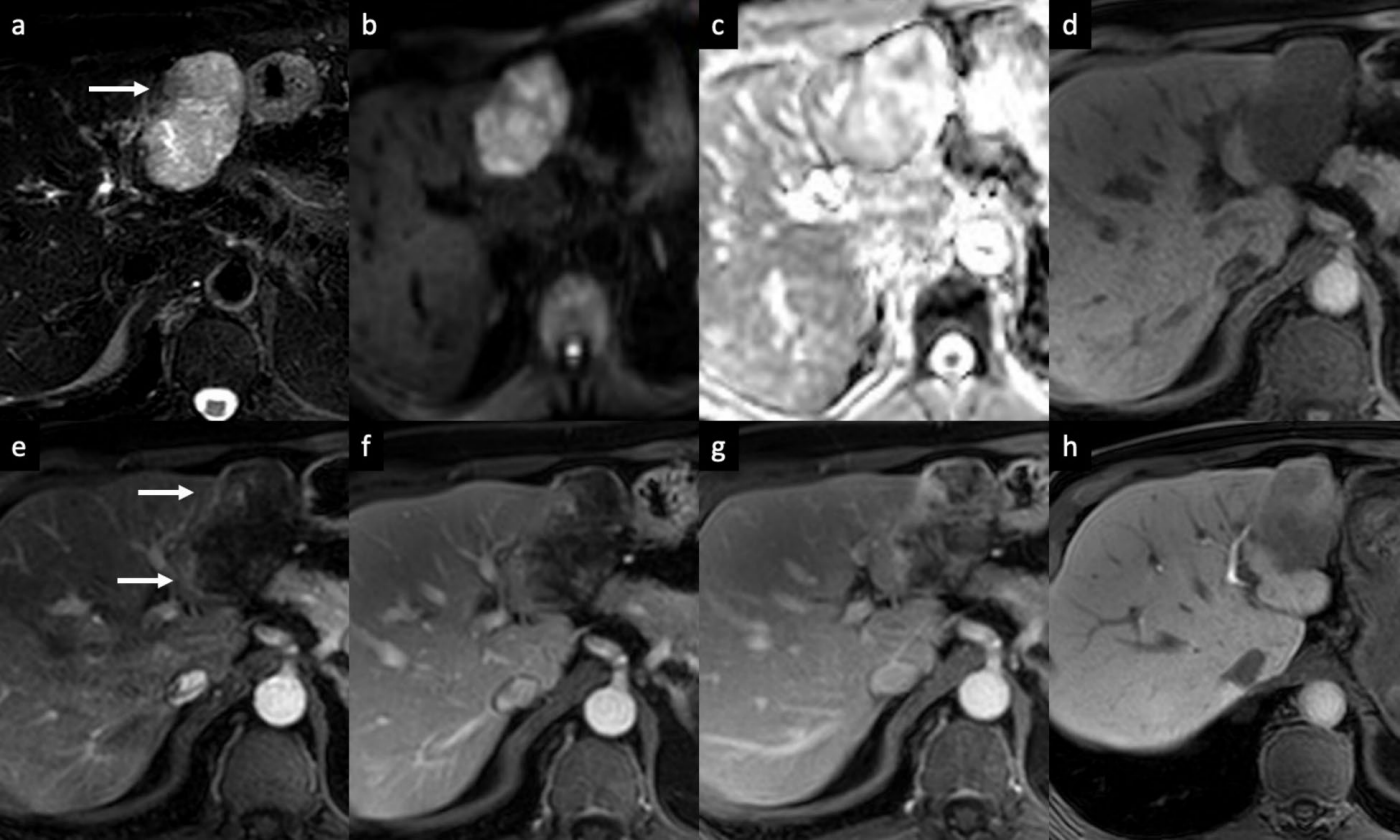
Sclerosed hemangioma in a 69-year-old man. MRI shows a focal liver lesion in the left lobe with heterogenous high signal intensity on axial fat-suppressed T2-weighted image (arrow, **a**), with high signal intensity on diffusion-weighted imaging (**b**), and high intensity on the apparent diffusion coefficient map (**c**). On contrast-enhanced MR images the lesion shows low signal intensity on unenhanced T1-wighted image (**d**), and peripheral discontinuous enhancement on arterial phase (arrows, **e**), followed by a progressive centripetal enhancement on portal venous (**f**) and delayed (**g**) phases. On the hepatobiliary phase acquired 2 h after the administration of gadobenate dimeglumine contrast agent, an heterogenous low signal intensity is observed

Hemangioma is the most common benign tumor of the liver, with a reported incidence of 2–20% in the healthy adult population [3–6]. Hemangiomas are usually incidental findings in routine examination of asymptomatic patients. Giant hemangiomas are typically defined as being larger than 4–5 cm in diameter, and may be responsible for liver enlargement and abdominal discomfort as well as complications such as rupture and Kasabach-Merritt syndrome [3–6]. Sclerosed hemangiomas are rare and more common in cirrhotic liver, and they can be considered an end stage of cavernous hemangiomas with progressive fibrotic involution changes, such as thrombosis, necrosis, or calcification with near complete obliteration of the vascular spaces [7–10]. On dynamic contrast-enhanced cross-sectional imaging, the typical features of hemangiomas include a hypoattenuating/hypointense well-defined lesion, with round or lobulated margins, with typical peripheral discontinuous globular hyperenhancement on HAP and progressive centripetal fill-in pattern that parallels the blood pool on PVP and DP. In addition, typical characteristics on MRI are low SI on T1-weighted images (T1-WI), intermediate to high SI on T2-weighted images (T2-WI), high SI on high *b*-value diffusion-weighted imaging (DWI) and on apparent diffusion coefficient (ADC) map, and hypointensity on hepatobiliary phase (HBP) (Fig. 1) [4–7, 11, 12]. On gadoxetic acid (Gd-EOB)-enhanced MRI, a high-flow hemangioma could show a pseudo washout pattern on the transitional phase due to contrast uptake in the surrounding normal liver parenchyma, mimicking hypervascular tumors such as hepatocellular carcinoma (HCC) [13]. In case of giant hemangiomas, the enhancement is usually discontinuous and peripheral on HAP, and then progressive, centripetal but often incomplete during the PVP and DP [7]. On MRI, giant hemangioma may demonstrate central cleft-like areas that show lower intensity on T1-WI and higher intensity on T2-WI, compared to the remaining part of the lesion (Fig. 2) [7, 14]. The imaging features are closely correlated with the macroscopic appearance, and include the above-mentioned central cleft-like areas due to hemorrhage, thrombosis, extensive hyalinization, cystic degeneration and liquefaction, and internal septa due to fibrosis [7, 14]. Sclerosed hemangiomas may demonstrate a peripheral discontinuous nodular hyperenhancement on HAP, with minimal progressive centripetal fill-in in the PVP and DP, due to extensive fibrotic involution of the lesion. These fibrotic changes result into heterogeneity of signal intensity on T2-WI with areas of typical high SI and areas of unusually low SI which may represent regions of sclerosis and fibrosis, and homogeneous retention of contrast agent on delayed imaging (Fig. 3) [15–17]. The tumor shows a slightly hyperintense area on DWI with a high SI on ADC map, due to the presence of many hyalinized tissues with poor cellular and fibrous components [18]. This high ADC mean values (approximately 2.00 × 10^−3^ mm^2^/s) may be useful for distinguishing sclerosed hemangioma from other malignant liver tumors such as cholangiocarcinoma or metastases that usually show low ADC values due to restricted water diffusion from high cellular density [19, 20]. Other features suggestive of sclerosed hemangioma include geographic pattern, capsular retraction, decrease in size over time, early rim enhancement, nodular regions of intense enhancement as seen in typical hemangioma, and loss of previously seen regions of enhancement [21]. Although a combination of these findings may suggest a presumptive diagnosis, a biopsy should be performed to obtain a definitive diagnosis.

#### Hepatic angiomyolipoma

**Fig. 4:**
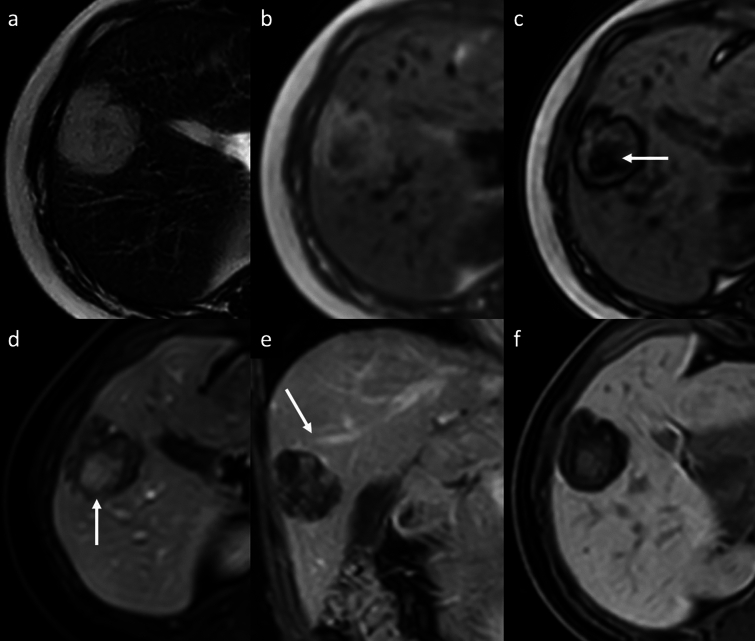
Hepatic angiomyolipoma in a 44-year-old woman. **a** Axial T2-weighted MRI shows a focal liver lesion arising in the right hepatic lobe with high signal intensity. Axial in-phase (**b**) and out-of-phase (**b**) gradient echo MR images show signal dropout at the center of the lesion, confirming the presence of fat in the lesion (arrow, **c**). Gadobenate dimeglumine-enhanced MRI obtained during the (**d**) arterial phase shows heterogenous low signal intensity of the lesion with small foci of peripheral discontinuous enhancement and a hypervascular central core (arrow, **d**). (**e**) Coronal T1-weighted image demonstrates the early venous drainage to the middle hepatic vein (arrow). The lesion shows a heterogenous appearance on (**f**) hepatobiliary phase acquired 2 h after the administration of contrast agent, displaying an hyperintense central core surrounded by a low signal intensity peripheral area

Hepatic angiomyolipoma (HAML) is a rare mesenchymal tumor belonging to a group of perivascular epithelioid cell tumors (PEComas), that is typically composed of varying proportions of proliferating thick-walled blood vessels, smooth muscle cells and mature adipose cells [22, 23]. HAML occurs usually as a solitary tumor in the non-cirrhotic liver and predominately in women, and about 5–10% of patients with tuberous sclerosis also have HAML [22]. It is usually asymptomatic and discovered incidentally [22, 23]. The imaging appearance is highly variable due to various proportions of the different tissue components. On CT, HAML typically appears as homogeneous or heterogeneous low-density, well-defined, lesion without a capsule and with a fat component [24, 25]. Dynamic contrast-enhanced CT shows remarkable hyperenhancement of the soft-tissue components with prominent central vessels on the HAP and sustained enhancement during the PVP, and, occasionally, lack of significant enhancement in the delayed phase depending on the component of the tumor tissue [24, 25]. On MRI, fat is hypointense on both fat-suppressed T1-WI and T2-WI, and the differential diagnosis can be facilitated by spectral spatial fat-saturation pulses or chemical-shift imaging [26, 27]. However, a considerable proportion of HAMLs (38.9–50%) may not have detectable intratumoral fat [24], and these lipid-poor type may therefore be easily misdiagnosed [24–27]. On extracellular contrast-enhanced MRI, persistent enhancement maintained for up to several minutes after the contrast agent injection is one of the features distinguishing HAML from HCC, even though 61.1% of the HAMLs may appear hypointense on PVP (Fig. 4) [28, 29]. The presence of enhanced tumor vessels in the peripheral portion of the tumor formed a peripheral enhancing rim and early draining veins on HAP, observed in 80–100% and 80–83.3% of cases of HAML respectively, can be helpful features for distinguishing HAML from HCC, with high specificity but low sensitivity [28]. Furthermore, the presence of tumor capsule on PVP or DP can be an important clue to suggest the possibility of HCC rather than HAML, because this feature was found in only 11% of HAMLs, in contrast to 50% of HCCs [24, 28, 29]. On DWI, about 17% of the HAMLs show isointensity, whereas the remaining 83% of the HAMLs also show high signal intensity [28, 29]. The enhancement profiles of HAML on Gd-EOB-MRI may overlap with those of HCC, except for the PVP; hypointensity during the transitional phase is reported in 88–92% of the HAMLs, and the tumors do not show any contrast uptake on HBP [29, 30]. Kim et al. [31] demonstrated that the HBP of Gd-EOB-MRI is the most beneficial sequence in discriminating HAMLs from HCCs; in this study HAMLs more frequently showed homogeneous hypointensity during the HBP images (83%), whereas only 41% of HCCs showed homogeneity on HBP images (*p* = 0.018). So, although diagnosis may be suggested by imaging, misdiagnosis in patients with HAML occurs frequently due to its highly variable presentation. Final confirmation with biopsy and histopathological examination remains crucial in this rare lesion [32–34]. The majority of HAMLs are clinically benign, and conservative follow-up may be recommended [33–36]. However, rare cases of aggressive changes including growth in size, recurrence after surgical resection, metastasis, and invasive growth pattern into the parenchyma and along the vessels have been reported, mostly in epithelioid type HAML [35]; in this context a prompt surgical resection is essential for better prognosis of this tumor (Table 1)*.*

**Table 1:** Focal liver lesions with discontinuous peripheral enhancement and their typical imaging features on contrast-enhanced CT and MRI

Observations	Typical imaging features on CT and MRI
Cavernous hemangioma	Well-defined lesion with round or lobulated margins Peripheral discontinuous nodular enhancement and progressive centripetal fill-in pattern that parallels the blood pool on PVP and DP Marked hyperintensity on T2-WI Hyperintense on high *b*-values DWI and on ADC map Pseudo washout pattern on the TP of Gd-EOB-MRI
Giant hemangioma	Lesion larger than 4–5 cm in diameter Peripheral discontinuous nodular enhancement and gradual centripetal incomplete fill-in pattern on PVP and DP Central cleft-like areas hypointense on T1-WI, hyperintense on T2-WI Complications such as rupture and Kasabach-Merritt syndrome
Sclerosed hemangioma	Peripheral discontinuous nodular enhancement with ultra-delayed centripetal enhancement Heterogeneity of SI on T2-WI due to sclerosis and fibrosis Hyperintense on high *b*-values DWI and on ADC map Geographic pattern, capsular retraction, decrease in size over time
HAML	Well-defined solitary lesion without capsule in non-cirrhotic liver Fat component hyperintense on both fat-suppressed T1-WI and T2-WI APHE with central vessels with sustained enhancement on PVP Peripheral enhancing rim and early draining veins on HAP Hypointensity on TP and on HBP
Hepatic peliosis	Focal or multiple lesions without mass effect on adjacent vessels Central “vessel-like” enhancement on HAP, with centrifugal or centripetal enhancement pattern on PVP Multiple T1- and T2-hyperintense intralesional hemorrhagic foci “Halo-like appearance” on Gd-EOB-MRI
Abscesses	Single or multiple well-defined lesions surrounded by peripheral transient perfusion disorders “Layered-wall appearance” with early inner wall rim APHE that persists on DP and progressive delayed enhancement of the outer layer “Cluster sign” “Double target sign” on T2-WI, restricted diffusion
HEH	Solitary or multiple lesions involving the peripheral regions of the liver Nodular or irregular discontinuous peripheral enhancement with progressive of the central fibrous stroma on PVP and DP T2-shine-trough effect and target appearance on DWI and on HBP “Lollipop sign’’ Capsule retraction and intralesional calcifications may be present
Primary hepatic angiosarcoma	Multifocal large lesion with heterogeneous internal architecture “Flame-shaped” peripheral discontinuous enhancement, progressive centripetal and delayed enhancement that parallels the blood pool “Reverse haemangioma centrifugal pattern”, with central enhancing foci expanded towards the periphery of the lesion Diffusion restriction of solid portions Spontaneous intratumoral hemorrhage, extracapsular tumor rupture and hemoperitoneum, extrahepatic metastasis
iCCA	Large lesion with irregular lobulated margins Early peripheral rim enhancement, progressive and delayed central enhancement with peripheral washout Targetoid appearance on DWI and on HBP Capsular retraction, dilatation and thickening of the intrahepatic ducts, vascular encasement, satellite nodules, intrahepatic metastases

*PVP* portal venous phase, *DP* delayed phase, *DWI* diffusion-weighted imaging, *ADC* apparent diffusion coefficient, *Gd-EOB-MRI* gadoxetic acid-enhanced magnetic resonance imaging, *HAML* hepatic angiomyolipoma, *TP* transitional phase, *HEH* hepatic epithelioid hemangioendotelioma, *iCCA* intrahepatic cholangiocarcinoma, *HBP* hepatobiliary phase

#### Hepatic peliosis

**Fig. 5:**
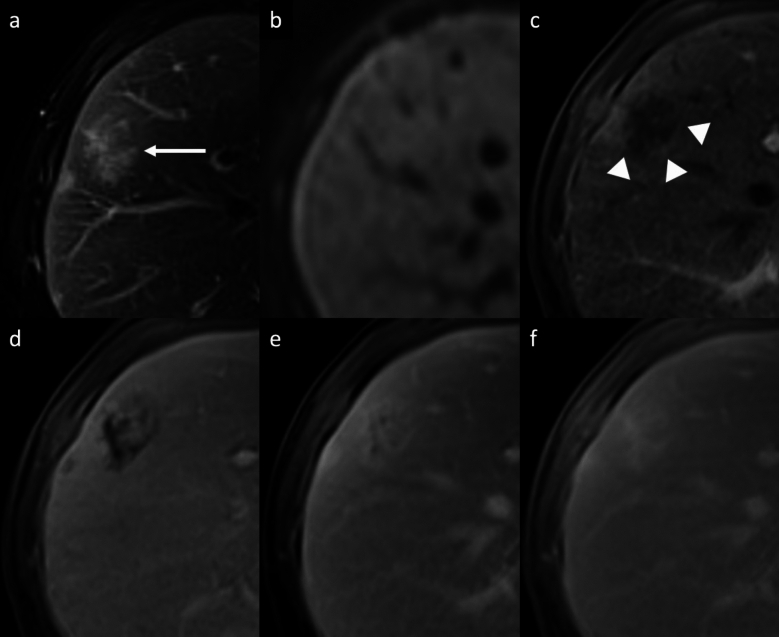
Hepatic peliosis in a 35-year-old woman with sinusoidal obstruction *syndrome. ***a** Axial fat-suppressed T2-weighted MRI shows a subcapsular focal liver lesion in the right lobe (arrow) with heterogeneous high signal intensity, and with an isointense signal on diffusion-weighted imaging (**b**). Extracellular contrast agent-enhanced MRI demonstrates a mild peripheral discontinuous enhancement on arterial phase (arrowheads, **c**), followed by a progressive and centrifugal enhancement on portal venous (**d**) and delayed phases at 3′ (**e**) and 5′ (**f**), showing a central focus of contrast pooling. Biopsy of the lesion confirmed the diagnosis of hepatic peliosis

Hepatic peliosis is a rare vascular nonneoplastic condition due to obstruction of the hepatic venous outflow at the level of the sinusoids, leading to dilation and breakdown of sinusoidal borders, hepatic veins, and liver plates and subsequent cystic necrosis [36, 37]. Patients often do not have symptoms but occasionally present with hepatomegaly, portal hypertension, hepatic failure, ascites, or severe abdominal pain due to hepatic rupture and hemoperitoneum [37, 38]. Typically, peliosis involves the entire liver, although cases of focal peliosis have been reported [39]. The typical imaging feature is focal or multiple irregularly shaped blood-filled lacunar spaces hypoattenuating on unenhanced CT scan, with a variable enhancement pattern: the lesions often show tubular (“vessel-like”) enhancement with central contrast medium accumulation (“target sign”) on AP, with centrifugal enhancement on PVP [37–39]. However, they may show a centripetal enhancement pattern, similar to that seen with cavernous hemangiomas. On MRI, lesions are usually hyperintense on T2-WI and hypointense on T1-WI, with multiple T1- and T2-hyperintense hemorrhagic foci (Fig. 5) [37–39]. On Gd-EOB-MRI, the lesion may show a central area of contrast agent uptake and this feature should be considered as the spared normal hepatocyte area (“halo-like appearance”), and it may be helpful in distinguishing hepatic peliosis from malignant ones [40]. Furthermore, lack of mass effect on adjacent hepatic vessels helps differentiate these lesions from other hepatic tumors. If diagnosed properly, the condition may resolve after appropriate treatment and cessation of potential causative drugs such as steroids, oral contraceptives, tamoxifen, and methotrexateand toxins that have been suggested as possible etiological factors [41].

### Infectious

#### Liver abscess

**Fig. 6:**
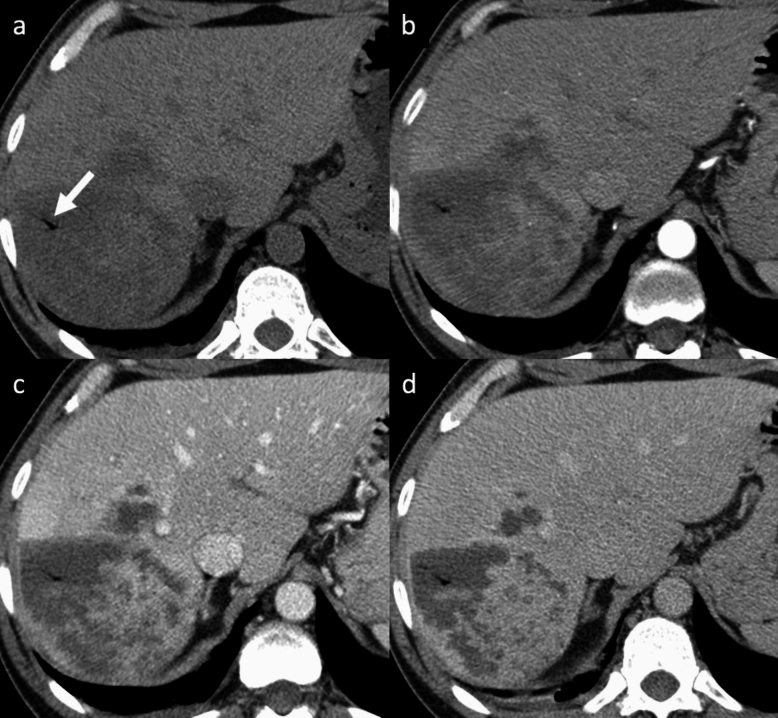
Liver abscess in a 48-year-old man with fever, jaundice and abdominal pain. **a** Axial unenhanced CT shows a hypoattenuating mass with irregular internal septa in the right hepatic lobe. Pneumobilia is visible inside the lesion (arrow, **a**). Contrast-enhanced CT shows a thick peripheral enhancement on arterial phase (**b**), that persists and progresses on portal venous (**c**) and delayed (**d**) phases

Abscesses can be the result of hematogenous dissemination of gastrointestinal infections via the portal vein or disseminated sepsis via the hepatic artery [42, 43]. Bile infection, favored by duct obstruction from various etiologies (stones, neoplasms, and strictures), biliary stents and bilio-digestive anastomosis, hepatic infection by continuity, and superinfection of pre-existing hepatic lesions, are other routes of liver abscesses [42, 43]. The typical clinical presentation includes fever, abdominal pain, nausea, leukocytosis, slightly elevated total bilirubin and aminotransferase levels, hypoalbuminemia. Amebiasis are relatively common parasitic diseases of the liver. At imaging, an amebic abscess usually presents as a solitary, rounded, well-defined cystic lesion with attenuation values that indicate the presence of complex fluid, with an enhancing thick wall of about 3–15 mm; the central abscess cavity may show septations and/or fluid-debris levels [44–46]. Pyogenic abscesses can be solitary or multiple, depending on the origin, with the ones with a biliary origin being usually multiple on both liver lobes and the ones coming through the portal vein being usually solitary and involving the right lobe [44–46]. In case of pyogenic abscesses, air within the lesion may occur in about 20% of cases, occasionally creating an air-fluid level. A typical imaging appearance of pyogenic abscesses is the double-target sign characterized by a central low-attenuation fluid-filled area surrounded by a high-attenuation inner ring and a low-attenuation outer ring; the entire lesion is usually surrounded by segmental geographic or peripheral transient perfusion disorders, identified as regions with APHE and isoattenuation on PVP and DP [44–46]. In case of multiple small pyogenic abscesses, they may tend to coalescence into a single large abscess cavity, leading to the so-called “cluster sign” [44–46]. However, in some cases the two entities are nearly undistinguishable. Capsular hyperintensity, ill-defined hyperintense areas, lesions with a tracklike appearance, and nodular areas at the liver may be seen at MR imaging as an inflammatory response, migration route, and fibrosis [44–46]. On MRI, abscesses show a central low SI on T1-WI, and central high SI on T2-WI, although the signal intensity may vary depending on the proteinaceous content; DWI shows hyperintensity high *b*-values and hypointensity on ADC map [44–46]. Although abscesses usually appear to be fluid collections, they may also have a more solid appearance (Fig. 6), mimicking primary or secondary hepatic tumors, such as intrahepatic cholangiocarcinoma (iCCA) or desmoplastic adenocarcinoma metastases. Areas of segmental discontinuous peripheral enhancement and perilesional edema surrounding organizing abscesses, or associated findings of malignancy (e.g. capsular retraction, biliary duct dilatation, lobar or segmental atrophy), are additional features that may help narrow the differential diagnosis between these entities. In some cases, differentiation may not be possible and aspiration/biopsy must be performed [44–46].

## Malignant lesions and lesions with malignant potential

### Vascular

#### Hepatic epithelioid hemangioendothelioma

**Fig. 7:**
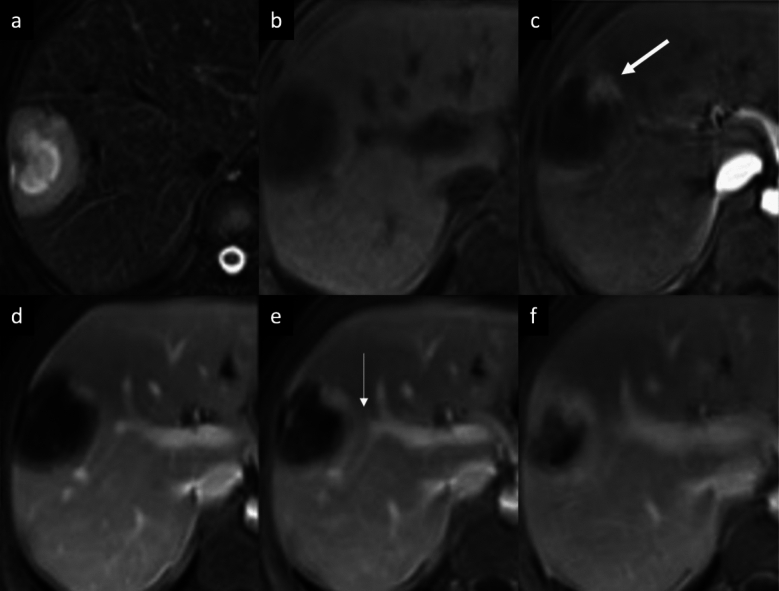
Hepatic epithelioid hemangioendothelioma in a 38 year-old woman. **a** Axial fat-suppressed T2-weighted MRI shows a subcapsular focal liver lesion in the right lobe with a three-layered target appearance. The high signal intensity of the lesion’s core is surrounded by a thin hypointense ring with a peripheral slight hyperintense halo. Capsular retraction also could be seen. The lesion shows a low signal intensity with a target appearance also on unenhanced T1-weighted MRI (**b**), with a central area of low signal intensity, surrounded by a thin slight hyperintense irregular ring with a peripheral hypointense halo. Extracellular contrast agent-enhanced MRI demonstrates a peripheral globular discontinuous enhancement on arterial phase (arrow, **c**), that minimally increases on portal venous (**d**) and delayed phases at 3′ (**e**) and 5′ (**f**). The typical ‘‘lollipop sign’’ is also visible on contrast-enhanced MRI, with portal veins entering and terminating in the periphery of the lesion (thin arrow, **e**)

Hepatic epithelioid hemangioendotelioma (HEH) is a rare vascular tumor of mesenchymal origin [47, 48]. The etiology is unknown; however, it may be associated with oral contraceptive use, exposure to polyethylene, trauma or viral hepatitis [47, 48]. Patients often have nonspecific symptoms, and one-third of patients presents extrahepatic lesions at the time of initial diagnosis; tumor marker levels are usually within normal limits [47, 48]. HEH may appear as solitary lesion or as hypodense nodules on unenhanced CT scan, that frequently coalesce and form larger confluent masses, with a propensity to involve the peripheral regions of the liver and to extend to the liver margin [47–49]. Nonspecific findings such as retraction of the liver capsule and of intralesional calcifications may be present [50, 51]. Contrast-enhanced dynamic imaging may show nodular or irregular discontinuous peripheral APHE followed by progressive of the central fibrous stroma on PVP and DP (“black target sign”) that is observed in 86.7% of cases [49–52] **(**Fig. 7**)**. Some lesions are surrounded by a thin, non-enhancing hypodense rim caused by tumor invasion of hepatic sinusoids, venules, and small portal vein branches. On MRI, HEH shows heterogeneous low SI on T1-WI, moderately hyperintense peripheral rim (due to the presence of proliferating tumor cells) and a markedly hyperintense central area on T2-WI (central stroma with necrotic areas and calcifications) [49–52]. On high *b*-value DWI, most of HEH lesions show a “target appearance” with a core of high SI, an intermediate thin ring of low SI, and a peripheral hyperintense rim, corresponded to a ring appearance on the ADC map as well with peripheral low ADC halo and high ADC core (consistent with T2-shine-through effect) [50–52]. Because HEH has the tendency to spread within the portal and hepatic vein branches, another specific finding is the ‘‘lollipop sign’’, a combination of the well-defined tumor mass on enhanced images (the candy in the lollipop) and the adjacent occluded vein (the stick) [53]. On Gd-EOB-MRI hepatobiliary phase HEH nodules usually appear completely hypointense with respect to the surrounding liver or may show an internal enhancement surrounded by a hypointense halo with an overall target appearance in 28.5% of cases [54]. Those signs are specific findings of HEH but they can also be seen in other entities, such as iCCA, abscesses, and liver metastases from various primary cancers (i.e., breast and colon cancer). Definitive diagnosis requires histopathologic confirmation [55].

#### Primary hepatic angiosarcoma

**Fig. 8:**
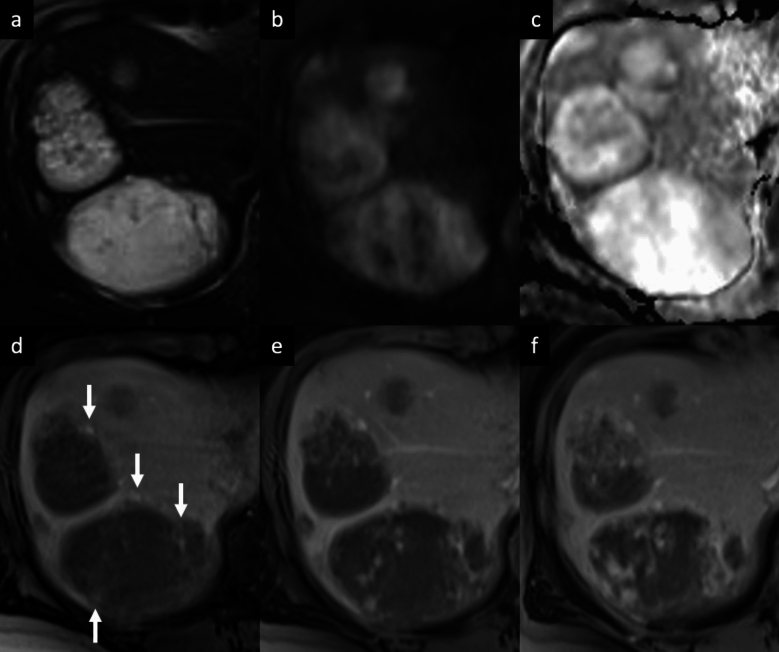
Hepatic angiosarcoma in a 35-years-old woman. **a** Axial fat-suppressed T2-weighted MRI shows multiple focal liver lesions in the right lobe with heterogeneous intermediate-to-high signal intensity. **b** On diffusion-weighted image the lesions demonstrate a heterogenous appearance, with mixed presence and absence of moderately hyperintense areas, displaying a heterogenous intralesional pattern on the apparent diffusion coefficient map (**c**). Extracellular contrast agent-enhanced MRI shows irregularly shaped and peripherally located foci of discontinuous enhancement on arterial phase (arrows, **d**), with an irregular flame-shaped both peripheral and central enhancement on portal venous phase (**e**) that progresses without a complete fill-in on delayed phase (**f**)

Primary hepatic angiosarcoma is very rare malignant and rapidly progressing vascular tumor, and it is the most common primary mesenchymal malignancy of the liver [37, 56]. Primary hepatic angiosarcomas sometimes mimic other multiple hepatic tumors of vascular endothelial cell origin, and the majority of patients present with nonspecific symptoms and multifocal growth in the liver as well as extrahepatic metastasis, leading to poor prognosis [37, 56]. Hepatic angiosarcomas typically manifest as multifocal, although in some cases it can may form a single large lesion [57]. On contrast-enhanced CT, typical features include multiple well-circumscribed lesions hypoattenuating on unenhanced scan, with heterogenous enhancing pattern, including early peripheral discontinuous nodular APHE (“flame-shaped pattern”), followed by progressive centripetal enhancement on PVP, or delayed enhancement in case of predominant fibrosis [57–59]; the attenuation follows blood pool. Most of cases show a “reverse hemangioma centrifugal pattern”, with central enhancing foci expanded towards the periphery of the lesion, while a minority of cases demonstrated complete flash-filling on the arterial phase, with blood pool matching on DP, which more closely mimicked benign hemangiomas [57–59]. The tumor may show a spontaneous intratumoral hemorrhage as well as an increased risk of hemoperitoneum from extracapsular tumor rupture [57–59]. MRI may demonstrate various features reflecting the heterogeneity of histopathological composition of the tumor which may contain areas of necrosis, fresh and old hemorrhage, fibrosis, and hyalinization [57–59]. Lesions with a heterogeneous internal architecture and predominantly solid portion may show intermediate-to-high SI on T2-WI, whereas in case of predominantly dilated vascular spaces they show strong SI on T2-WI like cavernous hemangioma; the hemorrhagic foci within the lesions appear as increased SI on T1-WI and decreased SI on T2-WI [57–60] (Fig. 8). On high *b*-value DWI, lesions with predominantly dilated vascular spaces may correspond to low cellularity with no diffusion restriction, whereas diffusion restriction is observed in lesions with predominantly solid portions corresponding to high cellularity [60]. In the context of underlying cirrhosis, the more relevant considerations in the differential diagnosis might include iCCA, cHCC-iCCA and multifocal HCC, but the lack of delayed washout and the absence of vascular invasion would argue against the latter. In noncirrhotic patients, the differential diagnosis should include peliosis hepatis, metastases (e.g. neuroendocrine tumors) [37].

### Biliary

#### Intrahepatic cholangiocarcinoma

**Fig. 9:**
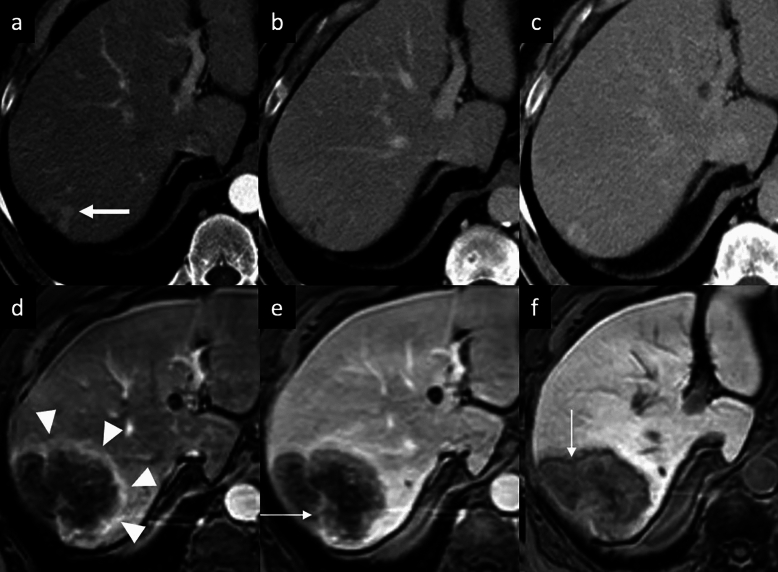
Intrahepatic mass-forming cholangiocarcinoma in a 74-year-old man with past medical history of prostatectomy who presented mild elevation of liver enzymes. **a**–**c** Contrast-enhanced CT exam shows a small subcapsular focal liver lesion on the right lobe, displaying a peripheral enhancement on arterial phase (arrow, a), that progresses on **b** portal venous and **c** delayed phases. On gadoxetic acid-enhanced MRI follow-up at 3 years the lesion increases in size and it shows a peripheral rim enhancement on arterial phase (arrowheads, **d**), with central intralesional enhancement on portal venous phase (thin arrow, **e**). **f** On hepatobiliary phase the tumor displays a “targetoid appearance”, with a central cloud-like hyperintensity surrounded with a low signal intensity peripheral rim (arrow, **f**)

**Fig.10:**
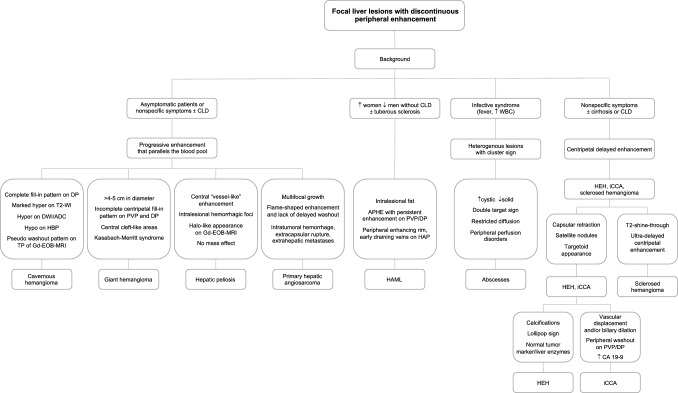
Proposed diagnostic algorithm flowchart for distinguishing between different focal liver lesions with discontinuous peripheral enhancement. *CDL* chronic liver disease, *PVP* portal venous phase, *DP* delayed phase, *DWI* diffusion-weighted imaging, *ADC* apparent diffusion coefficient, *HBP* hepatobiliary phase, *TP*  transitional phase, *Gd-EOB-MRI* gadoxetic acid-enhanced magnetic resonance imaging, *APHE* arterial phase hyperenhancement, *EHE* epithelioid hemangioendothelioma, *iCCA* intrahepatic cholangiocarcinoma, *HAML* hepatic angiomyolipoma

iCCA is the most common primary non-HCC malignancy in non-cirrhotic liver and manifests with different growth patterns, resulting in a broad spectrum of radiologic manifestations [61]. The mass-forming type is the most common form of iCCA [61]. On CT, it classically appears as a large low attenuation lesion with irregular lobulated margins, with early peripheral rim APHE, progressive centripetal enhancement and peripheral washout on PVP and DP [61–64]. The degree of enhancement of the tumor on the DP image is closely related to the amount of interstitial space in the fibrous stroma; this imaging finding has been reported in 81.8% of tumors with severe fibrosis [65], and it can be helpful feature for distinguishing iCCA from hemangioma, that typically appears isodense to the surrounding liver parenchyma on DP images [66]. Other common imaging findings include capsular retraction, dilatation and thickening of the intrahepatic ducts around the tumor, vascular encasement by the tumor, satellite nodules, and intrahepatic metastases; internal hemorrhage is rare due to prominent desmoplastic tumor stroma [61–64]. On MRI, iCCA appearance depends on the proportion of fibrosis, necrosis and mucin. Typically, iCCA shows low-to-moderate SI on T2-WI and low SI on T1-WI [67, 68]. iCCA may shows the “necrosis imaging sign” defined as a persistent, nonenhancing defect with either high SI or low SI on the T2-WI. DWI shows a “target sign” on high *b*-value images associated with peripheral hypointensity and central hyperintensity on ADC map [67, 68]. This “targetoid appearance” is also seen on HBP of Gd-EOB-MRI, which indicates a central cloud-like hyperintensity due to retained contrast material in fibrotic stroma surrounded with a low- SI peripheral rim (“EOB-cloud enhancement”) (Fig. 9) [68–70]. On Gd-EOB-MRI, iCCA may shows a pseudo washout pattern because of progressive background liver enhancement and no enhancement of the lesion [65–69]. Gd-EOB-MRI may aid in the diagnosis of iCCA because of increased lesion conspicuity and better delineation of intrahepatic daughter nodules (Fig. 10) [67–71]. Some atypical enhancement patterns are frequently seen, such as atypical homogeneous APHE in a well-differentiated tumor with less central necrosis reported in 29–46% of cases [72, 73], APHE and washout (especially in chronic liver disease or liver cirrhosis they may mimic HCCs), APHE without washout (the absence of washout in the DP may be helpful to avoid a false HCC diagnosis) [74, 75]. Mucinous subtype of iCCA may show marked hyperintensity on T2-WI and centripetal enhancement pattern, but it should be distinguished from a hemangioma on the basis of its continuous irregular peripheral enhancement, as opposed to the stronger and globular enhancement of the latter. In iCCA, peripheral rim APHE is the most frequently observed and the most sensitive LI-RADS feature (56.5–82.8%), followed by targetoid HBP on Gd-EOB-MRI (25.9–43.5%), and delayed central enhancement (24.2%) [76, 77]. Observations with a targetoid or infiltrative appearance, with marked diffusion restriction and necrosis in patients who meet the LI-RADS population criteria are likely to represent iCCAs, and in the assessment of patients with LR-M lesions serum tumor markers such as CA 19–9 may be helpful in diagnosing iCCA [78]. For instance, the final diagnosis of LR-M observations requires a histopathologic confirmation before treatment [76–78].

### Diagnostic approach to focal liver lesions with discontinuous peripheral enhancement

Considering the high prevalence of hemangiomas, in case of focal liver lesions with discontinuous peripheral enhancement, radiologists should look for imaging features that allow a likely diagnosis of hemangioma, including a typical peripheral discontinuous globular arterial phase enhancement with progressive centripetal fill-in pattern that parallels the blood pool. If the typical contrast-enhanced pattern is missing, imaging features of fibrotic changes may suggest a sclerosed hemangioma, whereas a large liver lesion with discontinuous peripheral enhancement and internal areas of hemorrhage, necrosis, cystic degeneration and liquefaction or fibrosis, are findings consistent with the diagnosis of giant hemangiomas.

However, several additional uncommon vascular mesenchymal neoplasms with peripheral discontinuous enhancing pattern may be considered on the basis of patient clinical presentation, multifocality, and imaging findings, such as hepatic epithelioid hemangioendothelioma and primary hepatic angiosarcoma. In patients with a hypervascular and hemorrhagic mass or multiple liver lesions, characteristic hematologic abnormalities may suggest a diagnosis of primary hepatic angiosarcoma. Hepatic peliosis is not vascular mesenchymal neoplasm but can mimic the other progressively enhancing lesions. In case of other arterially enhancing vascular mesenchymal neoplasms, the presence of intralesional fat may be helpful to diagnose hepatic angiomyolipomas.

If these signs are missing, and the patient has a clinical history that indicates the presence of infection, a single or multiple well-defined lesions showing discontinuous enhancement and a layered-wall appearance may be suggestive of hepatic abscess. Lesions with early peripheral rim arterial phase enhancement and targetoid appearance, associated with capsular retraction, and dilatation/thickening of the bile ducts may be suggestive of intrahepatic cholangiocarcinoma.

## Conclusion

A broad spectrum of focal liver observations may show discontinuous peripheral enhancement. In the majority of cases, this pattern of enhancement is related to the presence of a lesion of a vascular origin. The most common focal liver lesion with discontinuous peripheral enhancement is represented by cavernous hemangioma. However, cavernous hemangioma may have atypical presentations and other lesions with different malignant potential may mimic the pattern of cavernous hemangioma. Common and uncommon differential diagnoses may be considered on the basis of patient specific clinical settings and imaging findings, including both benign and malignant conditions. Because of the heterogeneity and complexity of these various entities, familiarity with the pathogenesis, knowledge of patient history, laboratory data, and typical imaging features on CT and MRI can aid radiologists to narrow the differential diagnosis and guide appropriate patient management. Finally, a histopathological examination may be required to resolve challenging cases.
